# Blood loss from transverse versus longitudinal uterine incision in abdominal myomectomy: a randomized controlled trial

**DOI:** 10.1186/s12905-020-01113-3

**Published:** 2020-12-28

**Authors:** Alaa Elguindy, Hosam Hemeda, Mohamed Esmat Shawky, Mohamed Elsenity, Medhat Adel Elsayed, Ahmed Fahim, Khaled Afifi, Maii Nawara

**Affiliations:** Department of Obstetrics and Gynecology, Faculty of Medicine, Ain Shams University, Abbaseyya Square, Cairo, Egypt

**Keywords:** Myomectomy, Blood loss, Transverse uterine incision, Longitudinal uterine incision, Randomized controlled study

## Abstract

**Background:**

It is unclear whether transverse uterine incision is non-inferior to longitudinal incision during myomectomy with regard to bleeding. Our aim was to compare between transverse and longitudinal uterine incisions in myomectomy.

**Methods:**

A parallel randomized controlled single-blinded study in a university affiliated hospital, in the period between January 2017 and April 2018, in which 52 women candidates for abdominal myomectomy were randomized into transverse uterine incision or longitudinal uterine incision groups (26 in each group). Intraoperative blood loss (estimated directly by blood volume in suction bottle and linen towels and indirectly by difference between preoperative and postoperative hematocrit), operative time and postoperative fever were analyzed.

**Results:**

No statistically significant difference was found between transverse and longitudinal incisions regarding intraoperative blood loss (389.7 ± 98.56 ml vs 485.04 ± 230.6 ml respectively, *p* value = 0.07), operative time (59.96 ± 16.78 min vs 66.58 ± 17.33 min respectively, *p* value = 0.18), and postoperative fever (4% vs 8.33%, *p* value = 0.6).

**Conclusion:**

Transverse uterine incision does not cause more blood loss than longitudinal incision and is a reasonable option during abdominal myomectomy.

Trial registration: NCT03009812 at clinicaltrials.gov, registered January 2017

## Background

Uterine leiomymomas are considered a chief health issue, with around 235 million females affected throughout the globe, and up to 70% of females could be diagnosed with uterine myomas before menopause. On the other hand, symptomatic leiomyomas requiring management exist in only 25% of patients [1].

Myomectomy is the typical surgical management option for various women who suffer from myomas and require future childbearing or basically desire preservation of their uterus. Various surgical approaches for myomectomy are implemented in practice involving laparotomy, laparoscopy, and hysteroscopy [2, 3].

Myomectomy is considered a risky bloody procedure, and the most considerable morbidity correlated to it is major blood loss [4]. Being a reconstructive surgery, the goals of myomectomy operation include removal of the tumor mass without excessive blood loss, closure of the dead space without hematoma formation, ensuring a good-quality uterine scar that can withstand subsequent pregnancies without dehiscence, and minimizing postoperative formation of adhesions.

It has usually been proposed that vertical uterine incision was the most preferred as it causes less blood loss, being attributed to the fact that arcuate arteries run transversely from lateral to medial [4, 5]. However, radiological studies proved that the presence of the myoma disrupts the normal vascular design, so either transverse or longitudinal incision would transect the arcuate arteries [6].

We postulate that transverse uterine incisions might achieve a better scar quality. It is well established that classic longitudinal incisions are associated with weaker scars and higher incidence of subsequent uterine rupture compared to lower segment transverse incisions during cesarean delivery [7, 8]. Despite the unique process of uterine involution and remodeling after delivery [9], which hinders extrapolating outcomes of wound healing to myomectomy incisions; some correlations might prove some relevance. Relative strength of low transverse uterine scars was reported in preterm uteri with yet under-developed lower segment, compared to the classic longitudinal scars [10].

Thus, it might seem a rational option to perform a transverse uterine incision [11] if proven to be hemostatically equivalent to longitudinal incision. Unfortunately, up to our knowledge, no previous studies addressed this issue in open myomectomy.

The current research study aims to prove that transverse uterine incision is not inferior regarding intra-operative blood loss compared to longitudinal incision during myomectomy.

## Methods

### Study design

A parallel randomized controlled pilot study conducted at Ain Shams University Maternity Hospital in the period between January 2017 and April 2018, in which fifty-two cases were recruited from those attending the outpatient gynecologic clinic, who were candidates for myomectomy.

### Sample size calculation

Sample size was calculated using the Power & Sample Size Calculator®, setting the power (1−β) at 0.8 and the type-1 error (α) at 0.05. The primary outcome of the current study is the mean estimated intraoperative blood loss (IBL). Reviewing the literature revealed no direct comparison between vertical and transverse uterine incision for abdominal myomectomy. A trial comparing the two types of incision in *laparoscopic* myomectomy was, however, found. This latter trial showed that the mean values for estimated IBL in transverse versus vertical uterine incision in laparoscopic myomectomy were 110.5 ± 81.7 ml and 136.4 ± 108.5 ml, respectively [12]*.* Therefore, transverse uterine incision would be assumed to reduce the mean estimated IBL by almost 18.9%. A previous study showed that the average blood loss during conventional abdominal myomectomy (which utilizes vertical uterine incision) was 621 ± 121 ml [13]*.* The mean estimated IBL with transverse uterine incision would, therefore, be assumed to be 503 ml. Calculation according to these values, produces a minimal sample size of 17 women in each group. Considering an attrition rate of 35% (to compensate for drop-outs due to the limited surgical experience with the novel technique in difficult myomectomies), a total of 52 women were recruited.

### Recruitment

Criteria for inclusion in the study were as follows; single myoma measuring 5–10 cm in diameter, causing abnormal uterine bleeding, uterine pressure-related symptoms, infertility or recurrent pregnancy loss in women between 20 and 35 years of age, with BMI between 18.5–29.9 kg/m^2^. Candidates were excluded from the study if they had any of the following criteria; pregnancy, cases with bleeding tendency, prior laparotomies, cases having coexisting pelvic pathologies, as ovarian cysts. All candidates had a complete preoperative evaluation via clinical history taking, clinical examination and sonographic examination for confirmation of the exact site, size and number of uterine fibroids and to exclude any coexisting pelvic pathology, a venous blood sample for blood picture, liver and kidney functions as well as coagulation profile, as a part of anesthetic workup.

Convenience sampling was utilized. The process of recruitment and handling the study population during the course of the study is shown in the CONSORT flow diagram. Candidates were randomly assigned (with 1:1 allocation ratio) to one of two groups according to a computer generated sequence, distributed in sequentially numbered sealed opaque envelopes, allocated by the seventh author. Only the research candidates were blinded to the surgical technique.
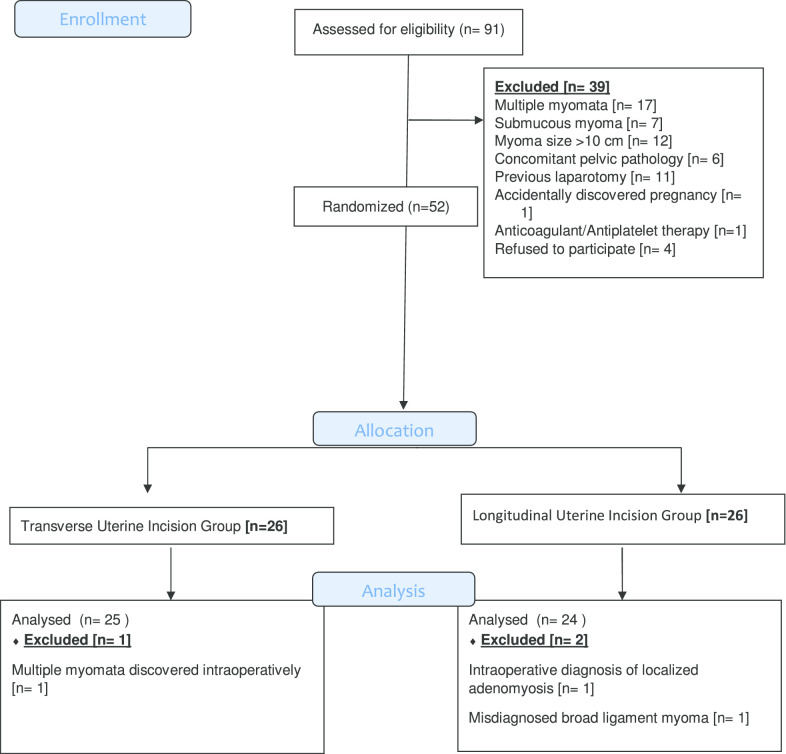


### Intervention

**Fig. 1:**
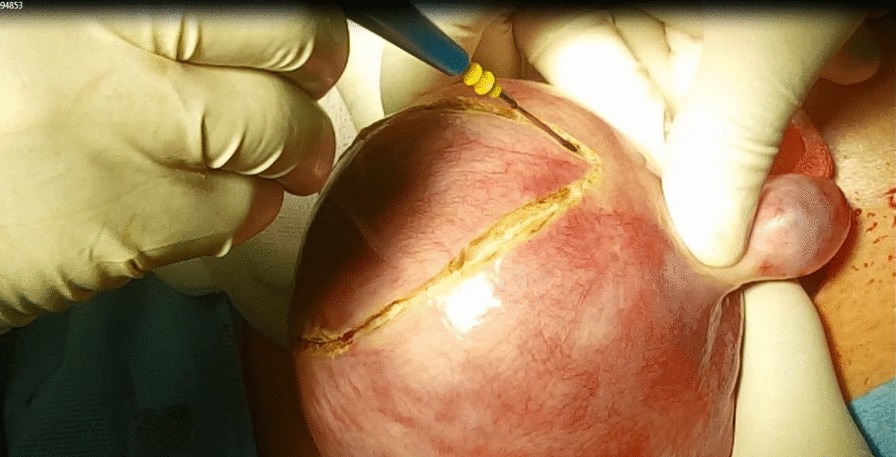
Transverse elliptical uterine incision

**Fig. 2:**
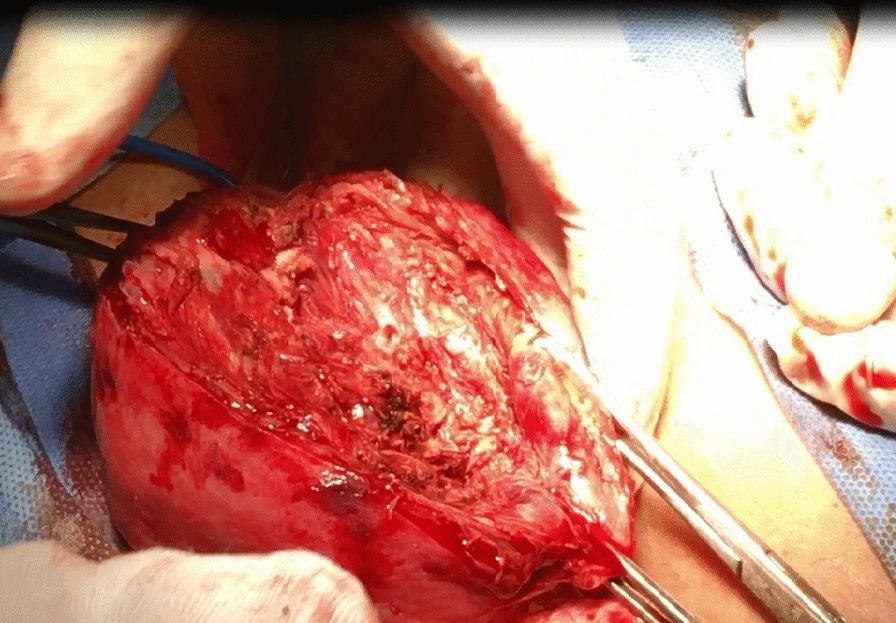
Transverse incision after removal of the myoma

**Fig. 3:**
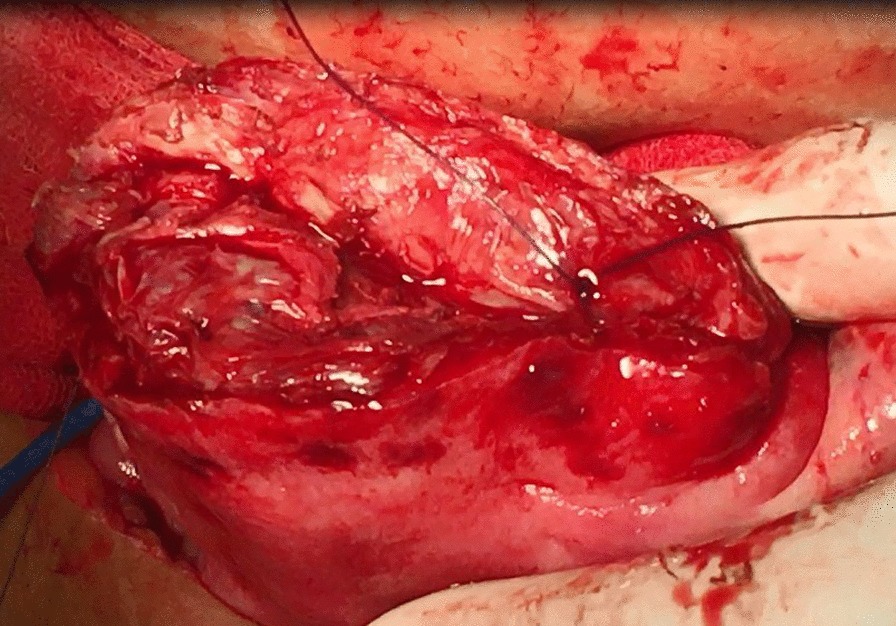
Closure of transverse incision

**Fig. 4:**
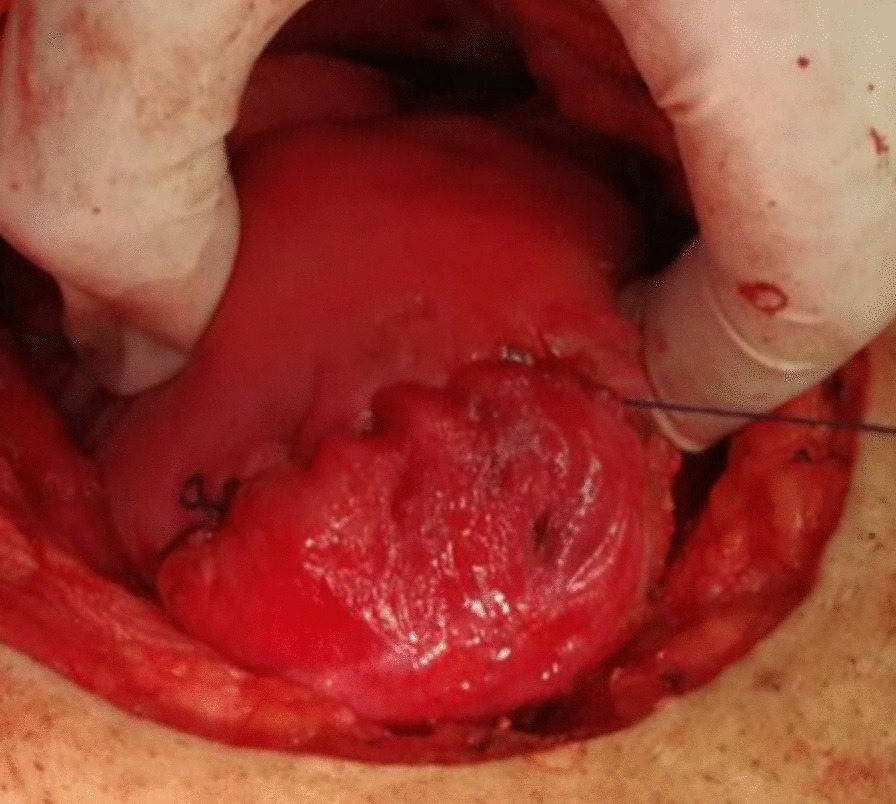
Transverse incision after closure

All candidates received general anesthesia and the abdominal cavity was accessed through a Pfannenstiel incision. Research group A included 26 women who were planned for transverse uterine incision in which a transverse elliptical uterine incision is accustomed within the pseudocapsule in a manner that its transverse diameter is almost centralized over the myoma with a slight anterior shift. Its length is 2 cm shorter than the maximum diameter of the myoma and ends well away from the tubal ostia. Breadth of the ellipse is fashioned so that its edges wouldn't be under tension or redundant after closure, usually the breadth equals one third of its length (Fig. 1). Tissue planes are bluntly and/or sharply dissected and the myoma is resected with the overlying ellipse of myometrium and pseudocapsule leaving a relatively smaller zone of dead space for closure (Fig. 2). Myometrium is re-approximated in two layers using absorbable suture (Fig. 3). The uterine serosa is sutured in a running fashion, exposing minimal suture material and raw area to minimize adhesion formation (Fig. 4). Research group B included 26 women who were planned for standard longitudinal uterine incision [4]. No vasopressin or any other hemostatics were used to avoid their confounding effect on blood loss. No peritoneal toilet was done and intra-peritoneal suction drains were left in all cases. IBL was estimated as follows**:** obtaining the volume difference of blood in suction bottle containers (in ml), obtaining weight difference of linen towels (in gm**)** [weight of soaked linen towels—weight of dry linen towels] [14]. These differences were estimated from the beginning of the uterine incision till its closure. IBL was also calculated by obtaining the difference between preoperative and 24 h postoperative hematocrit using the following formula:

## EBV x (Hi-Hf)/Hi

Where the Hi is the preoperative blood hematocrit, Hf is the postoperative one and EBV is the estimated blood volume***. ***The estimated blood volume could be calculated by multiplying weight times average blood volume which is around 65 ml/kg in females [15].

After exclusion of three patients (as shown in the CONSORT flow diagram), a total of 25 women in the Transverse Uterine Incision group and 24 women in the Longitudinal Uterine Incision group were eligible for statistical analysis.

Postoperatively, all women were subjected to postoperative follow-up of vital data and suction drains for 24 h, and received NSAIDs for analgesia.

### Research study outcomes

IBL as primary outcome, operative time and postoperative fever, defined as temperature of 38.5 °C in the first 48 postoperative hours and persisting more than 24 h [16, 17], as secondary outcomes.

### Statistical analysis

Conducted using SPSS for Windows version 20. Per protocol analysis was adopted to avoid dilution of treatment effect by excluded patients with surgically-difficult myomectomies.

Data was represented as mean ± SD (95% CI) for continuous parametric variables and median (IQR) for non-parametric ones. Difference between two unrelated groups was analyzed using the independent Student’s *t* test as well as the mean difference [MD] and its 95% confidence interval [95% CI] (for numeric parametric variables); Mann–Whitney’s U test (for numeric non-parametric variables); paired *t* test (for paired numerical parametric variables); chi-squared test and Fischer exact test (for categorical variables). Significance level was set at 0.05.

## Results

No statistically significant differences were found between women of both groups regarding age, body mass index or parity. Also there were no significant differences between women of both groups regarding frequency distribution of indications for myomectomy, myoma location according to FIGO sub-classification [18], mean myoma size, preoperative hemoglobin or hematocrit. (Table 1).

**Table 1:** Comparison between study groups regarding basic demographic characteristics, indication for myomectomy and myoma size, location, preoperative hemoglobin and hematocrit

	Transverse uterine incision group (n = 25]	Longitudinal uterine incision group (n = 24]	*p*
Age (Yrs) Mean ± SD (95% CI)	28.84 ± 3.77 (27.2–30.3)	29.04 ± 4.68 (27.0–31.0)	0.86^a^
BMI (Kg/m^2^) Mean ± SD (95% CI)	24.36 ± 2.85 (23.1–25.5)	23.73 ± 2.45 (22.7–24.7)	0.41^a^
Parity	3 (1–4)	3 (2–5)	0.29^b^
Indication for myomectomy			
AUB	16 (64%)	15 (62.5%)	0.76^c^
Pressure symptoms	5 (20%)	5 (20.8%)	
Infertility	1 (4%)	0 (0%)	
RPL	3 (12%)	4 (16.6%)	
Mean myoma size (mm) Mean ± SD (95% CI)	81.72 ± 14.03 (75.9–87.5)	82.29 ± 9.85 (78.1–86.4)	0.87^d^
Myoma localization			0.70^c^
Type 3	3 (12%)	2 (8.33%)	
Type 4	4 (16%)	4 (16.66%)	
Type 5	8 (32%)	4 (16.66%)	
Type 6	7 (28%)	10 (41.66%)	
Type 7	0	0	
Type 8	0	0	
Hybrid myomas (Type 2–5)	3 (12%)	4 (16.66%)	
Preoperative hemoglobin concentration (gm/dL) Mean ± SD (95% CI)	12.34 ± 0.97 (11.9–12.7)	12.63 ± 1.22 (12.1–13.1)	0.35^d^
Preoperative hematocrit (%) Mean ± SD (95% CI)	37.35 ± 3.01 (36.1–38.5)	38.25 ± 3.80 (36.6–39.8)	0.36^d^

AUB, abnormal uterine bleeding; RPL, recurrent pregnancy loss

^a^Analysis using unpaired t-test

^b^Analysis using Mann–Whitney U-test

^c^Analysis using chi squared test

^d^Analysis using unpaired t-test with Welch’s correction

^e^According to the Leiomyoma FIGO sub-classification system [18]

Directly-estimated IBL (based on weight difference of gauze and towels and suctioned blood volume) was lower in the transverse uterine incision group compared to the longitudinal uterine incision group (389.7 ± 98.56 vs 485.04 ± 230.6); though this difference failed to reach statistical significance. (*p* value = 0.07). Only one case needed postoperative blood transfusion, due to anemic symptoms in the longitudinal incision group after an estimated blood loss of 1000 ml, but that was not statistically significant. (*p* value = 0.48) (Table 2).

**Table 2:** Comparison between study groups regarding directly-estimated IBL, need for blood transfusion, postoperative hemoglobin and hematocrit drop and calculated blood loss

	Transverse uterine incision group (n = 25]	Longitudinal uterine incision group (n = 24]	*p*
Estimated IBL (mL) (95% CI) Mean ± SD (95% CI)	389.7 ± 98.56 (349.0–430.4)	485.04 ± 230.6 (387.6–582.4)	0.07^a^
Need for blood transfusion	0 (0%)	1 (4.16%)	0.48^b^
Hemoglobin concentration (gm/dL)			0.68^c^
Preoperative	12.34 ± 0.97	12.63 ± 1.22	
Postoperative	10.68 ± 1.02	10.13 ± 1.81	
Mean Paired	− 1.65 ± 0.71	− 2.50 ± 1.84	
Difference (95% CI)	(− 1.95 to − 1.36)	(− 3.28 to − 1.72)	
Hematocrit (%)			0.72^c^
Preoperative	37.35 ± 3.01	38.25 ± 3.80	
Postoperative	29.92 ± 2.88	28.37 ± 5.07	
Mean Paired	− 7.43 ± 2.12	− 9.87 ± 5.28	
Difference (95% CI)	(− 8.31 to − 6.55)	(− 12.10 to − 7.64)	
Calculated IBL (mL) Mean ± SD (95% CI)	311.78 ± 78.85 (279.2–344.3)	388.03 ± 184.55 (310.1–465.9)	0.07^d^

^a^ Analysis using unpaired t-test with Welch’s correction

^b^ Analysis using Fisher’s exact test

^c^ Analysis using repeated measure ANOVA test. Only *p* value for between-subject effect is displayed

^d^ Analysis using unpaired t-test

Postoperative repeated analysis of hemoglobin concentration revealed higher drop in hemoglobin concentration in the longitudinal uterine incision group in comparison to the transverse uterine incision group; though lacking statistical significance. (*p* value = 0.68) Similar findings were found on analysis of hematocrit values in the two groups (*p* value = 0.72). Calculated IBL (derived from difference between pre- and post-operative hematocrit values) between the two groups was higher in the longitudinal incision group, yet this difference did not reach statistical significance (*p* value = 0.07). (Table 2).

No statistically significant difference was observed between the transverse uterine incision group and the longitudinal uterine incision group regarding the operative time (*p* value = 0.18). (Table 3).

**Table 3:** Comparison between study groups regarding operative time, fluid in suction drain and incidence of postoperative pyrexia

	Transverse uterine incision group (n = 25]	Longitudinal uterine incision group (n = 24]	*p*
Total operative time (min) Mean ± SD (95% CI)	59.96 ± 16.78 (53.0–66.8)	66.58 ± 17.33 (59.2–73.9)	0.18^a^
Fluid in suction drain (mL) Mean ± SD (95% CI)	123.0 ± 37.79 (107.3–138.6)	113.5 ± 20.27 (104.9–122.0)	0.27^b^
Postoperative pyrexia	1 (4.0%)	2 (8.33%)	0.60^c^

^a^Analysis using Mann–Whitney U-test

^b^Analysis using unpaired t-test

^c^Analysis using Fisher’s exact test

There was no statistically significant difference as regards the amount of fluid lost in the suction drain or incidence of postoperative pyrexia (*p* value = 0.27, 0.60 consecutively). (Table 3).

## Discussion

Longitudinal midline uterine incisions have usually been adopted during myomectomy [4, 5]. They were assumed to be more hemostatic, because the anatomical studies of the distribution of myometrial blood vessels showed that arcuate arteries run transversely from lateral to medial, to join at the median plane. They give off radial arteries and spiral arterioles, which course centripetally towards the endometrium in also a transverse plane [19–21]. This transverse course would thus, at least theoretically, result in transection of more blood vessels by a longitudinal incision. In comparison, a transverse uterine incision will parallel their course, leading to the transection of a fewer number [22].

Moreover, suturing of a vertical incision places the sutures parallel to the course of the transversely running vessels, which hinders adequate ligation of all transected arteries and veins, together with their branches and tributaries respectively. On the contrary, suturing transverse incisions places the sutures perpendicular to them, and thus, provides an effective hemostatic seal that would surround all transected vessels [22].

However, all these assumptions were based upon anatomical studies of normal uteri. Few studies addressed the distortion of myometrial vascular anatomy by the growing myomas. Studies of the vascular pattern of myomatous uteri by injection, micro-radiographic and histologic techniques revealed that their vascular supply depends upon localized expansion of the normal myometrial vasculature, where displaced and distorted arcuate and radial arteries give off a number of small arteries that penetrate the myoma anywhere along its circumference [23]. They also revealed that the venous drainage of fibroids is sparse compared to the arterial supply, only few moderate-sized distended arcuate veins are occasionally seen. These distended venous channels contribute substantially to the heavy bleeding during myomectomy [23]. Angiographic study of the course of the arterial blood vessels encircling uterine myomas revealed that the neo-vessels, developing around them, do not follow the normal anatomy of myometrial vasculature. Instead, on the surface of myomas, blood vessels run mostly diagonally with an angle of 0–60° in 70% of cases. In addition, nearly 40% of the vessels crossed the myoma midline [6].

Based on these findings, it may be concluded that arterial vessels on the surface of myomas can be injured whatever the direction of uterine incision, yet a transverse uterine incision would be less traumatic to the transversely directed myometrial arteries, aretrioles and venous channels, and to the diagonally directed fibroid feeding vessels.

This theory was previously questioned by Morita and his colleagues in the setting of laparoscopic myomectomy. Transverse uterine incisions were associated with significant decline in IBL compared to longitudinal incisions (137.6 ± 88.1 vs. 235.8 ± 169.4 mL respectively). This decline was more evident in large myomas (> 7 cm in diameter); with an estimated blood loss 158.9 ± 87.1 mL in transverse incisions, compared to 362.3 ± 147.3 mL in longitudinal incisions. Whereas no statistically significant differences were found between both incisions in smaller myomas (< 7 cm) [12]. This seems logic, as the traumatic effect becomes more evident with the extension of the longitudinal incision causing more damage to the traversing vessels; in contrast to the extension of the transverse incision that runs almost parallel to the vessels. However, validation by further studies is needed, as the proposed effect of myoma size in the study of Morita is confounded by the large difference in operative time with myomas > 7 cm between longitudinal (165.4 min) and transverse incision (129 min), owing to the easier suturing technique of transverse incisions [12].

Despite the disparities between the laparoscopic and open approaches for myomectomy, in addition to preoperative treatment with gonadotrophin-releasing hormone analogs and intra-myometrial vasopressin injection in the study of Morita [12]; yet, the underlying surgical theory of transection of more transversely running vessels by longitudinal incisions might also be held true in case of open myomectomies.

In consistence with the same theory, the results of our study proved that transverse uterine incision resulted in lower volumes of IBL and postoperative drop of hemoglobin and hematocrit compared to longitudinal incision, although this difference did not reach statistical significance. It should be noted that only myomas < 10 cm in diameter were included in our study, with an average myoma size of approximately 8 cm.

We acknowledge the differences between open and laparoscopic approaches for myomectomy [24, 25], yet it is standard practice in our institute to perform myomectomy through the open approach for cases with our inclusion criteria.

The optimum myomectomy technique has always been a matter of debate, especially concerning measures to ensure optimum myometrial healing, considering the difficulty in the assessment of the obstetric quality of the resulting scar. The concept of pseudocapsule preservation in the intracapsular myomectomy technique has been introduced to preserve the neurovascular bundle and promote better healing [26].

Although based on a sound biological background, much of the assumptions of the impact of pseudocapsule preservation are still theoretical and derived by analogy to the effect of preservation of the neurovascular bundle during prostatectomy [27]. Reports of the reproductive outcomes of intracapsular myomectomy seem favorable [26]. However, up to our knowledge, no trials compared the obstetric quality of the scars of intracapsular myomectomy with those of the conventional surgical approach. Moreover, given the inevitable injury of the pseudocapsule during the incision to reach the myoma, no clear cut limits exist for the maximum accepted damage to the pseudocapsule. It is not known whether the whole pseudocapsule should be preserved or even a smaller part would be sufficient to supply the growth mediators postulated to stimulate myometrial healing.

Elliptical uterine incisions are designed to remove excess serosal and myometrial tissues overlying the maximum fibroid bulge, to avoid leaving behind large dead space after enucleation of the myoma. The idea of removal excess serosal and myometrial tissues is reported in the literature several times [28]. Especially in the setting of minimally invasive myomectomy, many surgeons advocate this incision to ensure obliteration of any dead space [29].

We adopted an elliptical incision, removing only a small part of devascularized serosa and pseudocapsule overlying the myoma within the boundaries of the ellipse, to minimize dead space and the amount of redundant tissue to be sutured, while sparing the rest of the pseudocapsule.

The mean blood loss in both research groups of our study lies in the average range of blood loss volume observed in previous studies, that indicated that mean blood loss during open abdominal myomectomy would be between 200–800 ml [13, 30–32].

The mean operative time ranged between 58–88.5 min in different studies using the standard longitudinal myomectomy incision [13, 33, 34], which matches our observed mean operative time in both research groups. The variation between different studies in calculated blood loss and operative time was correlated to the size and number of excised myomas.

In this study, only one case required blood transfusion in the longitudinal incision group representing 4% of cases, while none required in the transverse incision group, making no significant difference between both groups. Studies on average blood loss during myomectomy stated that blood transfusion was widely variable extending from 2 to 28% of cases [35].

Postoperative pyrexia following myomectomy has been linked to the development of myoma-bed hematoma [16], that may be followed by weakening of the developing scar [36]. In this study, postoperative pyrexia developed in one patient in the transverse incision group and in two patients in the longitudinal incision group. However, being a secondary outcome, our sample size is underpowered to detect a significant difference in the incidence of such uncommon complication.

By proving that blood loss is comparable in both incisions, it might be convincing to routinely adopt a transverse uterine incision during myomectomy as with cesarean section. Future research is required to investigate long term sequelae of transverse and longitudinal incisions during myomectomy regarding reproductive sequelae, incidence of uterine rupture in subsequent pregnancies and its impact on formation of pelvic adhesions.

Among the strengths of this study: (1) up to our knowledge, this is the first study to explore the effect of uterine incision direction in open myomectomy; (2) homogeneity of patients within the two groups regarding the number, size and site of myomas; (3) estimation of IBL both directly and indirectly. Limitations include: (1) although we tried to restrict the range of myoma sizes, we failed to statistically adjust blood loss to varying myoma size due to limited sample size; (2) different surgeons were involved in the study, although all had similar adequate experience.

## Conclusion

Blood loss from transverse uterine incision is comparable to longitudinal incision in open myomectomy.

## Data Availability

The data used to support our findings are available from the corresponding author if requested.

## Abbreviations

AUB: Abnormal uterine bleeding
BMI: Body mass index
EBV: Estimated blood volume
IBL: Intra-operative blood loss
RPL: Recurrent pregnancy loss
